# An expert-curated global database of online newspaper articles on spiders and spider bites

**DOI:** 10.1038/s41597-022-01197-6

**Published:** 2022-03-28

**Authors:** Stefano Mammola, Jagoba Malumbres-Olarte, Valeria Arabesky, Diego Alejandro Barrales-Alcalá, Aimee Lynn Barrion-Dupo, Marco Antonio Benamú, Tharina L. Bird, Maria Bogomolova, Pedro Cardoso, Maria Chatzaki, Ren-Chung Cheng, Tien-Ai Chu, Leticia M. Classen-Rodríguez, Iva Čupić, Naufal Urfi Dhiya’ulhaq, André-Philippe Drapeau Picard, Hisham K. El-Hennawy, Mert Elverici, Caroline S. Fukushima, Zeana Ganem, Efrat Gavish-Regev, Naledi T. Gonnye, Axel Hacala, Charles R. Haddad, Thomas Hesselberg, Tammy Ai Tian Ho, Thanakorn Into, Marco Isaia, Dharmaraj Jayaraman, Nanguei Karuaera, Rajashree Khalap, Kiran Khalap, Dongyoung Kim, Tuuli Korhonen, Simona Kralj-Fišer, Heidi Land, Shou-Wang Lin, Sarah Loboda, Elizabeth Lowe, Yael Lubin, Alejandro Martínez, Zingisile Mbo, Marija Miličić, Grace Mwende Kioko, Veronica Nanni, Yusoff Norma-Rashid, Daniel Nwankwo, Christina J. Painting, Aleck Pang, Paolo Pantini, Martina Pavlek, Richard Pearce, Booppa Petcharad, Julien Pétillon, Onjaherizo Christian Raberahona, Joni A. Saarinen, Laura Segura-Hernández, Lenka Sentenská, Gabriele Uhl, Leilani Walker, Charles M. Warui, Konrad Wiśniewski, Alireza Zamani, Catherine Scott, Angela Chuang

**Affiliations:** 1 Laboratory for Integrative Biodiversity Research (LIBRe), Finnish Museum of Natural History (LUOMUS), University of Helsinki, Helsinki, Finland; 2 Molecular Ecology Group (MEG), Water Research Institute, National Research Council of Italy (CNR-IRSA), Largo Tonolli 50, 28922 Verbania Pallanza, Italy; 3 CE3C – Centre for Ecology, Evolution and Environmental Changes / Azorean Biodiversity Group and Universidade dos Açores, Angra do Heroísmo, Azores, Portugal; 4 Albert Katz International School for Desert Studies, Ben-Gurion University of the Negev, Sede Boqer Campus, Beersheba, Israel; 5 Blaustein Institutes for Desert Research, Ben-Gurion University of the Negev, Sede Boqer Campus, Beersheba, Israel; 6 Colección Nacional de Arácnidos, Instituto de Biología, Universidad Nacional Autónoma de México (UNAM), Mexico City, Mexico; 7 Environmental Biology Division, Institute of Biological Sciences, College of Arts and Sciences and Museum of Natural History, University of the Philippines Los Banos, 4031 Los Baños, Philippines; 8 Centro Universitario de Rivera, Universidad de la República, Montevideo, Uruguay; 9 Lab. Ecotoxicología de Artrópodos Terrestres, Centro Univeritario de Rivera, Universidad de la República, Montevideo, Uruguay; 10 Laboratorio Ecología del Comportamiento, Instituto de Investigaciones Biológicas clemente Estable (IIBCE), Montevideo, Uruguay; 11 Ditsong National Museum of Natural History, PO Box 4197, Pretoria, 0001 South Africa; 12 Department of Zoology and Entomology, University of Pretoria, Private Bag X20, Hatfield, 0028 South Africa; 13 Freelance translator, Verbania Pallanza, Italy; 14 Department of Molecular Biology and Genetics, Democritus University of Thrace, Komotini, Greece; 15 Department of Life sciences, National Chung Hsing University, No.145 Xingda Rd., South Dist., Taichung City, 402204 Taiwan; 16 Department of Biology, Macelwane Hall, 3507 Laclede Avenue, Saint Louis University, St. Louis, MO 63103 USA; 17 Croatian Biospeleological Society, Rooseveltov trg 6, Zagreb, Croatia; 18 Program Sarjana, Fakultas Biologi, Universitas Gadjah Mada, Yogyakarta, Indonesia; 19 Insectarium de Montréal, Espace pour la vie, 4101, rue Sherbrooke Est, Montréal, Québec H1X 2B2 Canada; 20 Serket, Arachnid Collection of Egypt (ACE), Cairo, Egypt; 21 Erzincan Binali Yıldırım University, Faculty of Science and Arts, Biology Department, 24002 Erzincan, Turkey; 22 The National Natural History Collections, The Hebrew University of Jerusalem, Edmond J. Safra Campus, Givat Ram, Jerusalem, 9190401 Israel; 23 The Department of Ecology, Evolution and Behavior, The Hebrew University of Jerusalem, Edmond J. Safra Campus, Givat Ram, Jerusalem, 9190401 Israel; 24 Botswana International University of Science and Technology, Palapye, Botswana; 25 UMR CNRS 6553 Ecobio, Université de Rennes, 263 Avenue du Gal Leclerc, CS 74205, 35042 Rennes Cedex, France; 26 Department of Zoology and Entomology, University of the Free State, P.O. Box 339, Bloemfontein, 9300 South Africa; 27 Department of Zoology, University of Oxford, Oxford, OX1 3PS United Kingdom; 28 Department of Biological Sciences, National University of Singapore, 14 Science Drive 4, Singapore, 117543 Singapore; 29 Department of Biotechnology, Faculty of Science and Technology, Thammasat University, Rangsit, Pathum Thani 12121 Thailand; 30 Dept. of Life Science and Systems Biology, University of Torino, Via Accademia Albertina, 13 - 10123, Torino, Italy; 31 Unit of Conservation Biology, Department of Zoology, Bharathiar University, Coimbatore, 641046 Tamilnadu India; 32 National Museum of Namibia, Windhoek, Namibia; 33 5A Sagar Sangeet, SBS Marg, Mumbai, 400005 India; 34 Department of Biological Sciences, Ajou University, Suwon, Republic of Korea; 35 Research Centre of the Slovenian Academy of Sciences and Arts, Jovan Hadži Institute of Biology, Ljubljana, Slovenia; 36 University of Greifswald, Zoological Institute and Museum, General and Systematic Zoology, Loitzerstrasse 26, 17489 Greifswald, Germany; 37 Department of Natural Resource Sciences, McGill University, 21 111 Lakeshore Road, Sainte-Anne-de-Bellevue, Quebec, H9X 3V9 Canada; 38 Department of Biological Science, Macquarie University, Sydney, NSW 2122 Australia; 39 Mitrani Department of Desert Ecology, University in Midreshet Ben-Gurion, Midreshet Ben-Gurion, Israel; 40 BioSense Institute – Research Institute for Information Technologies in Biosystems, University of Novi Sad, Dr Zorana Đinđića 1, 21000 Novi Sad, Serbia; 41 National Museums of Kenya, Museum Hill, P.O. BOX 40658- 00100, Nairobi, Kenya; 42 School for Advanced Studies IUSS, Science, Technology and Society Department, 25100 Pavia, Italy; 43 Institute of Biological Sciences, Faculty of Science, University of Malaya, 50603 Kuala Lumpur, Malaysia; 44 Department of Animal and Environmental Biology, Federal University, Oye-Ekiti, Ekiti State Nigeria; 45 Te Aka Mātuatua School of Science, University of Waikato, Private Bag 3105, Hamilton, 3240 New Zealand; 46 Independent researcher, Toronto, Canada; 47 Museo Civico di Scienze Naturali “E. Caffi”, Piazza Cittadella, 10, I-24129 Bergamo, Italy; 48 Ruđer Bošković Institute, Bijenička cesta 54, 10000 Zagreb, Croatia; 49 Biodiversity Research Laboratory, Moreton Morrell, Warwickshire College University Centre, Warwickshire, United Kingdom; 50 Institute for Coastal and Marine Research, Nelson Mandela University, Port Elizabeth, South Africa; 51 Department of Entomology, University of Antananrivo, Antananarivo, Madagascar; 52 School of Biological Sciences, University of Nebraska-Lincoln, Lincoln, Nebraska 68588 United States; 53 Department of Biological Sciences, University of Toronto Scarborough, 1265 Military Trail, Scarborough, Ontario M1C 1A4 Canada; 54 Natural Sciences, Auckland War Memorial Museum, Parnell, Auckland, 1010 New Zealand; 55 Te Pūnaha Matatini, University of Auckland, Auckland, New Zealand; 56 Murang’a University of Technology, Department of Physical & Biological Sciences, P.O.Box 75-10200, Murang’a, Kenya; 57 Institute of Biology and Earth Sciences, Pomeranian University in Słupsk, Arciszewskiego 22a, 76-200 Słupsk, Poland; 58 Zoological Museum, Biodiversity Unit, FI-20014, University of Turku, Turku, Finland; 59 Department of Psychology, University of Tennessee, Knoxville, Tennessee USA; 60 Department of Entomology and Nematology, Citrus Research and Education Center, University of Florida, Lake Alfred, Florida USA

**Keywords:** Communication, Entomology, Ecology, Public health

## Abstract

Mass media plays an important role in the construction and circulation of risk perception associated with animals. Widely feared groups such as spiders frequently end up in the spotlight of traditional and social media. We compiled an expert-curated global database on the online newspaper coverage of human-spider encounters over the past ten years (2010–2020). This database includes information about the location of each human-spider encounter reported in the news article and a quantitative characterisation of the content—location, presence of photographs of spiders and bites, number and type of errors, consultation of experts, and a subjective assessment of sensationalism. In total, we collected 5348 unique news articles from 81 countries in 40 languages. The database refers to 211 identified and unidentified spider species and 2644 unique human-spider encounters (1121 bites and 147 as deadly bites). To facilitate data reuse, we explain the main caveats that need to be made when analysing this database and discuss research ideas and questions that can be explored with it.

## Abstract (summary)

**Table:** 

Measurement(s)	Newspaper articles on human-spider encounters
Technology Type(s)	Manual extraction
Sample Characteristic - Organism	Spiders (Arachnida: Araneae)
Sample Characteristic - Environment	Online
Sample Characteristic - Location	Global

## Background & Summary

Spiders have an unfortunate reputation. There are tales about massive infestations of false black widows shutting down entire schools; apocryphal stories of dangerous arachnids lurking under toilet seats of international airports; and urban myths of tiny spiders crawling into your mouth while you are asleep. Of course, these are just anecdotes, but they illustrate how, even today, arachnophobic sentiments permeate our society at all levels^1–4^. This is nothing surprising: arachnophobia is likely the most widespread fear related to animals^5^, with an estimated prevalence between 3.5–11.4% of the world population^6–9^. However, such a skewed perception towards the potential harm that spiders can cause humans contrasts with two facts. First, less than 0.5% of spider species can cause severe envenomation in humans^10^. Second, the habitat of these few potentially dangerous species rarely overlaps with that of humans, making dangerous human-spider encounters unlikely^11^. Since a limited number of fatalities due to spider bites have occurred in the past few decades^12–16^, the reasons behind our exaggerated perception of risk associated with spiders remain uncertain.

Despite gigantic leaps forward in cognitive science and neurology, we still do not know the exact reason why arachnophobia is so widespread. What we do know is that arachnophobic sentiments have a significant social and cultural component^17–19^. For example, a recent study suggested that arachnophobic behaviours may be reduced following an exposure to the superhero movie *Spider-Man*^20^. Likewise, it was suggested that ongoing urbanization is the key driver of the prevalence of disgust for insects and spiders, because exposure to animals in urban areas is less frequent^21^. Accordingly, it is reasonable to hypothesize that the public perception of spiders should be affected by how spider-related information is framed and circulated^2^, given that traditional media are particularly effective in disseminating knowledge by conveying messages quickly and reaching a broad audience^22^. From the belief in the role of bats as disease spreaders^23,24^ to the fear of being attacked by large carnivores^25,26^, the crucial role of traditional media in the construction and circulation of risk perception associated with wild animals is undisputed^27^. However, media representation of spiders is still a poorly studied subject: as far as we are aware, the only two available studies are focused on a local selection of news in Australia^28^ and Italy^2^.

**Fig. 1:**
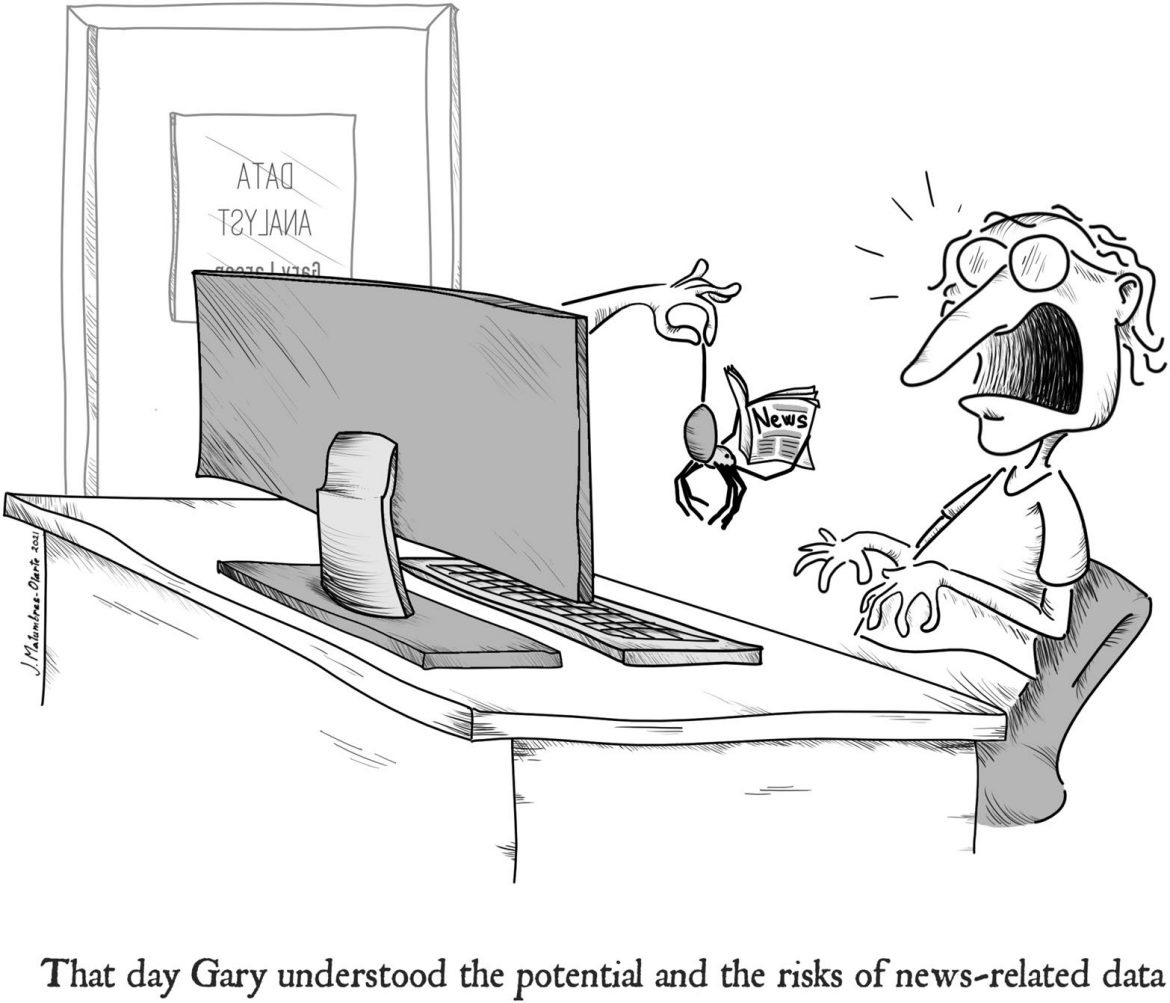
The potential of news articles as a source of data. This database offers a quantitative baseline to pursue research on the human-dimensions of spiders and their representation in the media. This research may include answering questions related to the cultural component of spider conservation, evaluation of people’s perceptions of spiders via opinion mining techniques, and generation of ecological insights, among others. Original illustration by Jagoba Malumbres-Olarte.

Intending to fill this gap, here we compiled an expert-curated global database on the coverage of human-spider encounters in online newspaper media and their accuracy and reliability over the past ten years (2010–2020) (Fig. 1). This database includes detailed information about the location of each human-spider encounter reported by the media, year of publication, and a quantitative characterisation of the content of each piece of news (presence of photographs of spiders and bites, number and type of errors, whether experts were consulted, and whether the content is sensationalistic or neutral). With this database, we hope to stimulate further research on the human dimension of spiders and their representation in the media.

## Methods

### Geographical coverage of the database

We aimed to compile a comprehensive database of global coverage of countries, languages and online newspapers. To this end, we put together a large network of spider experts to mine data in as many languages and countries as possible. We searched for news in 40 unique languages and covered the online press in 81 countries and all six continents where spiders can be found. Due to an uneven availability of experts, however, there is a bias in the database towards temperate regions (Europe and North America). African countries are the least represented.

### Temporal coverage of the database

We focused on newspaper articles published online between 2010 and 2020 (partial and uneven temporal coverage for 2020). Thus, the temporal span of our study mostly covered the advent of online journalism and the parallel diffusion of news through social media platforms^29^.

### Data mining protocol

We adapted the methodology of Mammola *et al*. (ref. ^2^) for retrieving news articles on human-spider encounters published in online newspapers in the target countries. To ensure that different authors in charge of different countries and languages adhered to an unequivocal data mining strategy, we began by preparing a general protocol for retrieving and extracting information from the news (Appendix S1). This protocol, shared with all authors, included: i) instructions for media report retrieval and data mining (see below); ii) a continuously updated list of Frequently Asked Questions discussing how to handle specific cases; iii) a description of the most common envenomation symptoms, which was used to standardize the assessment of the errors related to spider venom (see next section).

For each country and language, we carried out online searches in different languages with Google News (Appendix S1 – Figure S1), choosing multiple keyword combinations and the years between 2010 and 2020—this can be specified using the ‘Custom range’ tool in Google News (Appendix S1 – Figure S2). Note that for a few countries that were not available in Google News at the time of the search, such as Finland, Denmark, or Botswana, we performed searches directly in Google. We first searched for the words for ‘spider’ in each language, followed by the word ‘bite’ (e.g., “spider bite”). We repeated the search using the word ‘sting’ instead of ‘bite’, which is anatomically incorrect but often used^30^. We then repeated the search by changing the general word ‘spider’ with scientific and vernacular names of the main species perceived as “dangerous” in each country. These include species that are considered medically important (e.g., *Latrodectus* spp., *Atrax* spp., *Loxosceles* spp., and *Phoneutria* spp.; list of genera in Appendix S1 – Table S1) and/or widely feared (e.g., *Cheiracanthium* spp., *Lampona* spp., and *Steatoda* spp.; list of genera in Appendix S1 – Table S2). The list of species names used in searches for each country was tailored by each author, based on their expertise and knowledge of the spider fauna and the scientific literature on spider bites in the assigned country. We noted, however, that including additional search terms besides ‘spider bite’ and ‘spider sting’ yielded diminishing returns since these two broad keywords usually covered the vast majority of relevant news articles.

For each unique keyword search combination, we manually inspected all news up to the final available page in Google News, systematically collecting news articles referring to one or more purported encounters between humans and spiders. We included i) all news articles referring to a human-spider encounter (e.g., a family interviewed by a local newspaper about the spider they found in their house; a farmer bitten by a spider while working in the field; a person who was hospitalized following a spider bite); and ii) events that occurred either in the searched country or abroad (e.g., an Indian newspaper talking about a biting event that occurred in England). Note that we included all reports of human-spider interactions regardless of the likelihood of a spider actually being involved (e.g., a person claiming to have a spider bite when they likely had a skin infection instead). We disregarded: i) media items reporting general facts about spiders, venomous spiders, arachnophobia, spider-related research findings, and doctors’ advice about what to do in case of a spider bite; and ii) blog posts.

### Extraction of information for each news article

For each news article, we extracted the qualitative and quantitative information detailed in Table 1. We first reported basic information: a) URL; b) title; c) date of publication; d) newspaper name (or news outlet if not a traditional newspaper); e) newspaper type, broken down into “Traditional newspaper” (Official newspaper in a country, with both a printed and an online version), “Online newspaper” (online only newspaper), or “Magazine” (for magazines, tabloids, and similar); and f) newspaper circulation (regional, national, or international).

**Table 1:** Description of each column in the database.

Variable	Description
**ID**	A unique identifier for each media report. Note that the ID can be repeated when a news item includes multiple species or events.
**URL**	The link to the online media report. Because this was collected at the time of data mining, some URLs may not be working anymore.
**Language**	The language in which the media report is written.
**Country_search**	The country where the newspaper is published / where the search was conducted.
**Newspaper**	The newspaper in which the media report is published.
**Type_of_newspaper**	A generic description of the type of Newspaper. Levels: “Traditional newspaper” (Official newspaper in expert’s country, with both a printed and an online version), “Online newspaper” (online-only newspapers), or “Magazine” (for magazine, tabloids, etc.).
**Circulation**	The circulation of the Newspaper. Levels: “Regional”, “National”, “International”.
**d | m | y**	day, month, and year of publication of the media report.
**Title**	Article title (in the original language).
**ID_Event**	A unique ID for the human-spider encounter described in the media report, constructed by combining the Country_event, Location_event, and Year_event. An ID_Event can be repeated through the database when the same event was taken up by multiple newspapers.
**Year_event**	The year in which the ID_Event took place.
**Location_event**	The location (name of city/town/region) in which the ID_Event took place.
**Country_event**	The country in which the ID_Event described in the media report took place.
**Continent**	Continent in which the ID_Event described in the media report took place.
**lon | lat**	Coordinates (longitude, latitude) of the Location_event in decimal degrees (WGS84 reference system) (e.g., 7.47; 44.72). These were derived with Google Maps / Google Earth.
**lon2 | lat2**	Coordinates (longitude, latitude) of the Country_event in decimal degrees (WGS84 reference system) (e.g., 7.47; 44.72). These were derived with Google Maps / Google Earth.
**lon3 | lat3**	Coordinates (longitude, latitude) of the Country_search in decimal degrees (WGS84 reference system) (e.g., 7.47; 44.72). These were derived with Google Maps / Google Earth.
**Species**	The scientific name of the spider species involved in the ID_Event, as reported in the news item. If the species is not mentioned and/or impossible to infer from the text and figures, the notation “Gen sp” is used.
**Genus**	The genus of the spider involved in the ID_Event.
**Family**	The family of the spider involved in the ID_Event.
**Order**	The order “Araneae” (to which all spiders belong) is used unless the media report incorrectly assigned other organisms as spiders (e.g., harvestmen, camel spiders, insects).
**Bite**	Does the human-spider encounter result in a bite? 1 = yes; 0 = no.
**Death**	Does the human-spider encounter result in a deadly bite? 1 = yes; 0 = no.
**Figure_species**	Does the media report contain a photograph (or video content) of a spider species? 1 = yes; 0 = no.
**Figure_bite**	Does the media report contain a photograph (or video content) of a spider bite? 1 = yes; 0 = no.
**Expert_arachnologist**	Was an expert consulted/capable of identifying the spider involved in the ID_Event (arachnologist, entomologist, taxonomist, etc.)? 1 = yes; 0 = no.
**Expert_doctor**	Was a medical doctor or other similar medical professional consulted in the media report? 1 = yes; 0 = no.
**Expert_others**	Was any other ‘expert’ consulted in the news (e.g., a pest controller)? 1 = yes; 0 = no.
**Sensationalism**	Is the media report sensationalistic/overstated? 1 = yes; 0 = no.
**Taxonomic_error**	Does the article contain any taxonomic error? 1 = yes; 0 = no.
**Venom_error**	Does the article contain any error related to spider venom? 1 = yes; 0 = no.
**Anatomy_error**	Does the article contain any error related to the anatomy of spiders? 1 = yes; 0 = no.
**Photo_error**	Does the article contain any error in the photographs (or video content)? 1 = yes; 0 = no. Note that we used ‘NA’ if there was no photo present.
**Quality_check**	Was the article re-assessed (see section “*Data accuracy and curation*”)? Levels: “yes”, “yes*” (when a new entry was added as a result of the re-assessment), and “no”.
**Contributor**	The researcher(s) who collected the data associated with the specific media report.
**Notes**	Any other information related to the media report.

In the database, the R notation ‘NA’ is used for missing values. See main text for more information.

Then, we read the full article and scored the g) spider species identity based on the description in the news article, even if the attribution was incorrect based on our expert opinion. We reported species identity to the lowest taxonomic level possible based on the information in the article (typically species or genus level, but sometimes only family level). If the species identification was not provided in the article but it was possible to infer (e.g., referring to a species being identifiable from a picture or a report of a “widow spider” identifiable to species based on geography), we reported this identification in the database. To achieve standardization throughout the database, we converted all names to the closest valid scientific name, based on the most updated spider taxonomy^31^. We next recorded the h) type of event, broken down into “encounter”, “bite”, or “deadly bite”; i) year of the event; and j) location of the event (latitude and longitude in decimal degrees). We used Google Maps to obtain WGS84 coordinates of the approximate centre of the most precise geographic region named in the article (i.e., the country, province/state, or city/town where the encounter occurred). Finally, we recorded the k) presence/absence of any photograph of the spider [whether or not of the actual spider(s) involved in the encounter]; l) presence/absence of photographs of the bite (regardless of whether the bite is being reported); and m) presence/absence of an expert-opinion, broken down into the categories “arachnologist” (e.g., spiders experts, taxonomists, entomologists), “medical professional” (e.g., doctors, veterinarians), and “other expert” (e.g., pest controllers) (see ref. ^32^ for a discussion).

Since several news articles often covered the same event, we created an identifier for each unique event (ID_Event), by combining location, country, and year of the event (e.g., “London_UK_2018”).

We assessed the quality of each news article by recording the presence/absence of any of four types of errors in the text and figures:

i) errors in photographs/figures, when the photograph(s) of the species in the news article (if any) did not correspond to the species mentioned in the text, or when the attribution was not possible (e.g., blurry photographs);
ii) errors in systematics and taxonomy, like the common mistake of considering spiders insects^33^ or inaccuracies in terms of species names and in higher Linnaean taxonomic ranks (e.g., referring to tarantulas as a single species or the genus *Latrodectus* as a family);
iii) errors in the description of venom toxicity, symptoms of envenomation, and other physiological or medical aspects or terminology (e.g., stating only female black widows can be venomous or describing the venom of recluse spiders, which causes tissue necrosis, as “neurotoxic”; see Appendix S1 for more details); and
iv) errors in morphology and anatomy, such as the frequent “spider sting” instead of “spider bite”^30^, or errors in describing the number of legs or eyes.

Each error type was scored as present or absent, so we did not count cumulative errors of the same type in the same news article.

Finally, we evaluated the title, subheadings, main text, and photographs/video content of each news article and assessed it as overstated (sensationalistic) or not (neutral). Sensationalism in animal-related news articles is often associated with emotional words, expressions, and images^2,25,26^. Examples of titles of sensationalistic versus non-sensationalistic news articles focusing on the same event are: “Thousands of spiders ‘bleed out of the walls’ and force family from home” vs. “Home Infested With Brown Recluse Spiders in Missouri”. Throughout the database, frequent words associated with sensationalistic content were ‘alarm’, ‘agony’, ‘attack’, ‘boom’, ‘deadly’, ‘creepy crawly’, ‘devil’, ‘fear’, ‘hell’, ‘killer’, ‘murderer’, ‘nasty’, ‘nightmare’, ‘panic’, ‘terrible’, ‘terrifying’, and ‘terror’^2^, as well as magnifying adjectives that exaggerated any features of the encounter (e.g., body size^34–36^, hairiness^35^). However, the presence of one of these words did not necessarily result in an article being scored as sensationalistic. For example, articles that referred to spider species whose venom can be fatal without medical intervention (e.g., *Latrodectus* and *Atrax* spp.) as ‘deadly’ could be overall non-sensationalistic, whereas articles describing non-medically important spiders as ‘deadly’ were more likely to be scored as sensational.

## Data Records

### Database availability

The database is freely available in Figshare^37^. The database is provided both as a tab-delimited file (.csv) and as an excel file (.xslx). Description of columns is in Table 1 but also in the metadata file uploaded alongside the database in Figshare. Code to access the database in R environment^38^ and derive basic summary statistics and graphs shown in this paper is available in GitHub (see section “Code Availability”).

### Description of the database

**Fig. 2:**
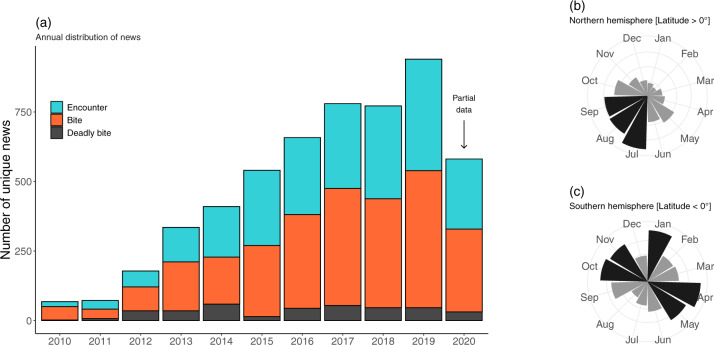
Temporal distribution of unique news articles. (**a**) Annual distribution of news articles by type of event (partial data for 2020). (**b**) Monthly distribution of news articles (cumulative of all years) in the northern hemisphere. (**c**) Monthly distribution of news articles (cumulative of all years) in the southern hemisphere—darker colours highlight months with the highest numbers of news articles.

In total, we collected 5348 unique news articles from 81 countries in 40 languages. The database has an uneven temporal coverage, with most news articles concentrating in recent years (Fig. 2a). There is also a seasonal pattern in the distribution of news articles. In the northern hemisphere, most news articles occur throughout the summer season (Fig. 2b), whereas the pattern is less clear in the south (Fig. 2c). The number of news items by countries varies by at least three orders of magnitude, from hundreds (United Kingdom: 865; United States: 537; Italy: 412; Russia: 395; France: 319) to a handful of news articles, or none (Table 2).

**Table 2:** Countries and languages for which no spider-related news articles were found.

Language (Country)	Expert in charge	Details
Basque (France, Spain)	Jagoba Malumbres-Olarte	No news article was found, both using Google or by searching directly the websites of the few newspapers in Basque.
English and Setswana (Botswana)	Tharina L. Bird; Naledi T. Gonnye	No news article was found, both using Google or by searching directly the websites of the local newspapers (n = 9). The lack of news was further confirmed by phone (see main text).
Galician (Spain)	Alejandro Martínez	No news article was found with direct search in Google and Google News.
Icelandic (Iceland)	Ingi Agnarsson (see section “**Acknowledgements**”)	No news article was found with direct search in Google and Google News. This lack of news is corroborated by personal communication with the most active entomologist in Iceland.
Montenegrin, Serbian, and Croatian (Montenegro)	Marija Miličić	No news article was found for Montenegro with direct search in Google and Google News.

**Fig. 3:**
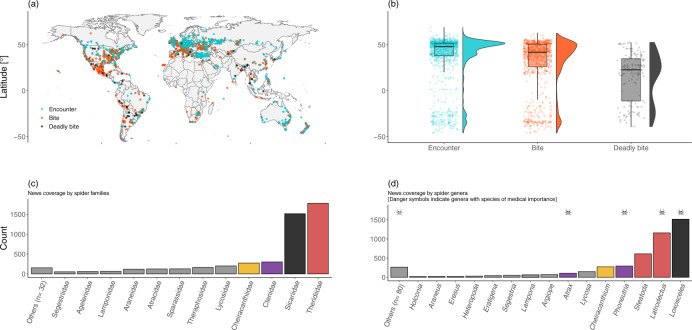
Geographical coverage of the human-spider encounters in the database. (**a**) Global distribution of event localities reported in the media report; due to the proximity of several localities, most points appear superimposed. (**b**) Latitudinal distribution of events. (**c**) News coverage by spider families. (**d**) News coverage by spider genera. Danger symbol marks genera with species of medical importance. In c–d, for the four most abundant families, colours represent families.

The database includes 6204 reports of human-spider encounters (corresponding to 2644 unique events) and 211 identified and unidentified spider species—note that a single news article may report about multiple human-spider encounters. Of these unique events, 1121 were reported by the news articles as bites, and 147 as deadly bites (Fig. 3a). The majority of reported encounters is concentrated at northern latitudes in the northern hemisphere (median latitude = 46.9°), whereas the median latitude of reported bites and deadly bites is further south (41.3° and 26.1°, respectively) (Fig. 3b).

**Fig. 4:**
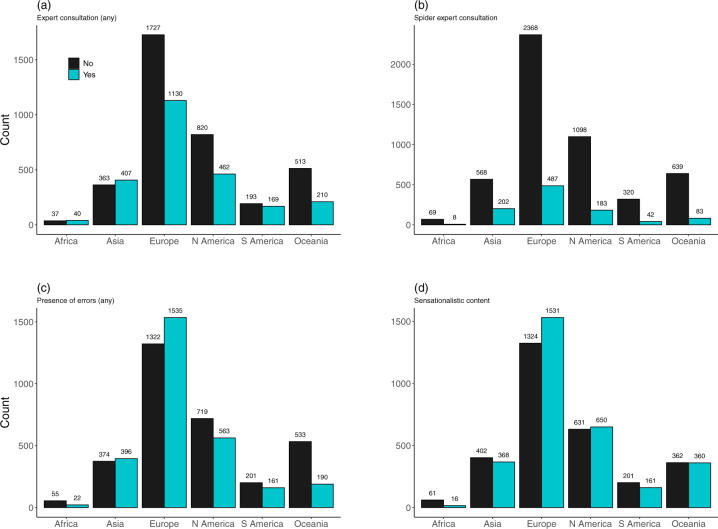
Content of news articles by continent. (**a**) Frequency of expert consultation in news articles (any type of expert). (**b**) Frequency of spider expert consultation (arachnologists, entomologists and similar) in news articles. (**c**) Frequency of errors in news articles (any type of error). (**d**) Frequency of sensationalistic versus non-sensationalistic news articles.

The presence of comments from experts consulted for articles about human-spider encounters varies substantially across countries and continents (Fig. 4a). Spider experts were only rarely consulted (Fig. 4b). One or more error types are present in 47% of news articles (Fig. 4c), although the frequency of different types of errors is variable (data not shown). Also, 43% of news articles were assessed by experts as sensationalistic; the frequency of sensationalistic versus non-sensationalistic news varied substantially by continent (Fig. 4d).

### Even zeros matter

For a few countries, we found no online news articles reporting human-spider encounters (Table 2). An informative case is Botswana, where the authors in charge performed an in-depth investigation to explain the lack of relevant results. Since Google News does not work for Botswana, they carried out the initial search in Google, which yielded no relevant results using any combination of keywords. To exclude the possibility this result was an artefact due to the searching tool, they repeated the search directly on the websites of the nine Botswana newspapers; once again, this search yielded no positive results. Finally, they phoned each of these nine newspapers individually. Six newspapers (Sunday Standard, The Midweek Sun, The Patriot, The Weekend Post, Mmegi, and Daily News) explained that their online presence is very recent and that the content placed online remains very selective. Thus, there were no human-spider encounters reported online. Conversely, they could not reach two newspapers by phone (Botswana Gazette and Botswana Guardian) and a third (The Voice Newspaper) declined to provide any information. This suggests that the search strategy is reliable in detecting absence of news articles, and these absences can be considered in analyses as ‘true zeros’^39^.

## Technical validation

### Data accuracy and curation

To increase the accuracy and internal consistency of our database, and given subjectivity in the assignment of certain values (e.g., sensationalism, errors), we re-assessed news for most articles in English (N = 1719; 80%) and some of the other most common languages based on the availability of native speakers (French, 53%; Italian, 88%; Spanish, 48%). The column “Quality_check” in the database indicates whether this re-assessment was performed for a given article. We assigned a pair of authors to a subset of these language-based datasets so they could independently re-examine and score articles that were previously mined by the original contributor. The re-assessors scored the articles in the same manner as described previously (see section “*Extraction of information for each media report”*) and compared their individual datasets with each other. Discrepancies in scores were discussed and compared with the original dataset to reach a consensus on final scores.

We estimated the rate of agreement between two re-assessors via Cohen’s kappa statistic^40^, calculated for all the scores of variables that may imply a degree of subjectivity in the assessment. We derived the confidence intervals using variance estimate^41^. We carried out this analysis only on English news (Table 3).

**Table 3:** Cohen’s kappa coefficients^40^ and confidence intervals^41^ for independent scoring of the same news articles. .

Variable	Cohen’s kappa (Confidence interval)	Possible reason for the discrepancy
Bite	0.99 (0.98–1.00)	In a few cases, it was not clear from the article description whether the biting event occurred or not.
Deadly_bite	0.94 (0.90–0.97)	In a few cases, it was not clear from the article description whether a fatality was attributable to the spider bite.
Figure_species	0.96 (0.94–0.97)	A photo may be overlooked for some articles filled with Ads or in presence of anti-spam filters. Also, some of the raters did not scored the presence of photos in video link.
Figure_bite	0.98 (0.96–0.99)	
Expert_arachnologist	0.94 (0.91–0.96)	The assessment of all these variables implies a certain degree of subjectivity. See Appendix S1 for more details.
Expert_doctor	0.91 (0.87–0.94)	
Expert_others	0.87 (0.83–0.90)	
Sensationalism	0.89 (0.87–0.91)	
Taxonomic_error	0.88 (0.84–0.92)	
Venom_error	0.90 (0.87–0.92)	
Anatomy_error	0.87 (0.80–0.94)	
Photo_error	0.85 (0.80–0.90)	

Cohen’s kappa statistic ranges from –1 to 1; values above 0.8 indicate very high to near perfect agreements among scorers. We performed this analysis only for rescored English news (N = 1719).

### Limitations in using the database

Users of the database must be aware of the following limitations:

i) The data collected here refer to online journalism only. The database does not cover the representation of spiders in the printed versions of traditional newspapers;
ii) Because Google’s search algorithm varies by country and user, the relationship between the number of published news articles and the number of results returned is likely not consistent. For example, the total number of news articles in China is unreliable due to the restrictions imposed by the government on Google. Consequently, we recommend against comparing absolute numbers of news items across countries, but rather always using relative numbers (e.g., proportion of errors, proportion of spider bites versus encounters, relative frequency of a given species in the press); and
iii) Likewise, any temporal trend must be interpreted with caution because news publishers can occasionally remove pieces of news from the Google News index or simply delete old news. The probability of this happening increases with time and thus may be partly responsible for the apparent increase in the volume of news articles over time in this database (Fig. 2a).

## Usage Notes

This database allows users to investigate questions related to the social dimension of spiders and the psychology of arachnophobia, but also contemporary problems in ecology^42,43^ and conservation biology^44^. To stimulate the use of the database, we discuss what we believe are some important avenues of research—while being aware that many other questions and patterns await to be explored^45^. Note that some of these research questions have already been briefly introduced in Mammola *et al*. (ref. ^2^), but here are expanded on and contextualized within the framework of a global-scale database.

### Comparison among countries and through time

Some of the first questions that come to mind are about the reasons underlying the disparity in the quality of news across regions and countries^2^, namely what are the main ecological, cultural, and/or social factors that explain the observed patterns? This dataset could be used to test the hypothesis that socio-economic factors, demographic features, level of education or literacy and/or cultural values affect the quality and taxonomic bias of spider-related news in a given country. For example, the relative number of reports of (presumed) bites in relation to encounters and quality of news articles may be greater in those countries/regions with either a high number of medically important species that can seriously harm humans (e.g., South America, Australia) or with a high species diversity (e.g., Brazil). Alternatively, an opposite pattern could emerge due to the paradoxically high prevalence of arachnophobia in areas with few or no dangerous spider species (e.g., the UK^46^). All these questions can be directly answered, among other ways, by summarizing relative values by country (e.g., proportion of errors, proportion of spider bites versus encounters) and by relating these variables with country-level indicators^47^.

### Comparison with other animal groups

The protocol for data mining discussed here is effective and inexpensive, and thus can be adapted to other cases. This would allow for comparisons of the types of errors and the levels of sensationalism across multiple taxa, including other venomous animals (e.g., bees, scorpions, wasps, snakes, and jellyfish) and answering questions such as:

i) Is there a relationship between taxon-specific features (distribution, diversity, adaptations, dangerousness, interactions with humans) and the content and quality of the media articles referring to them?
ii) Does a negative representation by the traditional media translate to a lower prioritization and fewer measures for conservation^48^? Conversely, does such sensationalism heighten public interest in nature, biodiversity monitoring, and invasive species?

### Link between traditional and social media

Social media have changed the way news information is framed and circulated^49^, including biodiversity-related content. In a recent study set in Italy, we found that the volume of newspaper articles shared on Facebook has increased substantially in recent years and that sensationalistic and overstated news stories about human-spider encounters are more likely to be shared^2^. Using the URL associated with each news article, one could directly analyse the shares on different social media platforms to answer questions about the factors that drive the popularity of news online^22,50^. To better understand the opinions, sentiments, and subjectivity of people sharing these news reports, one could even use text mining algorithms to perform quantitative analyses (e.g., sentiment analysis) on the public comments posted in response to the news (see next section).

### Linguistic analysis

**Fig. 5:**
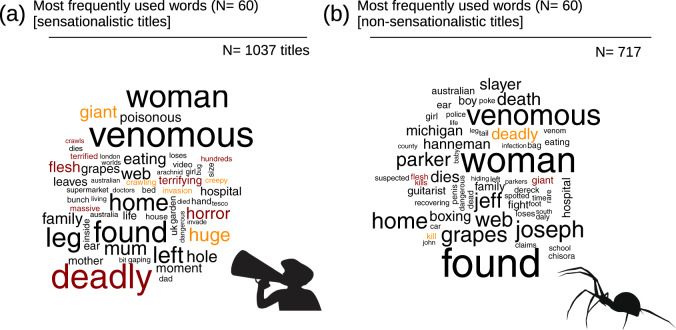
Usage of words in sensationalistic (**a**) versus non-sensationalistic (**b**) news articles titles. Word Clouds illustrating the most frequently used 50 words in the titles of English news articles. Common words (stop_words *sensu* ref. ^52^) and the terms used for online searching (e.g., ‘spider’, ‘bite’, ‘sting’, species names) are excluded from the analysis. Text size is proportional to the frequency of each word. Warm colours highlight words that appeal to emotions and are often associated with sensationalistic content. Original silhouettes by Irene Frigo, reproduced from ref. ^2^.

The title of each news article and other bodies of texts that can be automatically extracted using the URL of each news item (e.g., comments on the online article, comments in response to the shares of the article on social media) offer a large source of data in the domain of Sentiment Analysis or Opinion Mining—defined as the computational study of people’s emotions and attitudes toward a given topic^51^. A very simple example is provided in Fig. 5, where we used R text mining tools^52^ to compare the usage of words in sensationalistic versus non-sensationalistic titles of articles written in English. Inevitably, these kinds of analyses are more easily performed on news published in English because of the larger sample size.

### Source of iEcological knowledge

This database has the potential to generate ecological insights. Similar applications broadly fall within the domain of iEcology, an emerging research field that “[…] *seeks to quantify patterns and processes in the natural world using data accumulated in digital sources collected for other purposes*”^42^. The database of spider news provides data on > 200 spider species (Fig. 3d); for most of these species, we have recorded the coordinates of the locality where the human-spider encounter supposedly took place. Some of these records are clearly wrong (i.e., the species reported is not correct), but the presence of the supposedly encountered species could be verified by experts. In other words, the news reports could be used by experts (spider experts, in this case) to inspect areas where a given species may be present. Once a reliable database for a given species is cleaned, one could explore different ecological patterns.

In Italy, for example, the seasonal distribution of news articles on the Mediterranean black widow [*Latrodectus tredecimguttatus* (Rossi, 1790)] overlaps almost perfectly with the known phenology of the species^2^. Similar phenological insights may be checked for other species. Also, recent niche modelling studies have shown that internet-derived distribution data can be useful for mapping the predicted distribution of spiders (reviewed in ref. ^53^), especially species that are easily identified in the field or by photos^54–57^. For some of the most abundant species in our database (e.g., *Loxosceles* spp. and *Latrodectus* spp.), it is possible to compare whether the known distribution of a species overlaps with the predicted distribution based on the news. This way, one could quantify if the geographic and temporal distribution of human-spider encounters and bites reported in the news is related to the real distribution of a spider species, how and why this relationship varies among species, and what conservation-related or biosecurity measures may be necessary. Discoveries of spiders outside of their historical ranges may provide clues to pathways and new populations resulting from human-mediated dispersal events, such as western black widows (*Latrodectus hesperus* Chamberlin & Ivie, 1935) found in packages of grapes transported from California to Eastern North America and the UK, and reports of brown recluse spiders (*Loxosceles reclusa* Gertsch & Mulaik, 1940) in Michigan (USA).

## Supplementary information

Appendix S1

## Data Availability

The R code to generate analyses and figures is available in GitHub (https://github.com/StefanoMammola/Analysis_Global-Spider-News-Database).
