# Supplementary material for: An expert-curated global database of online newspaper articles on spiders and spider bites
Source: Sci Data. 2022 Mar 28;9:109. doi: 10.1038/s41597-022-01197-6 (PMC8960780; doi:10.1038/s41597-022-01197-6)
Supplement: Supplementary file 1 — Appendix S1 [file 41597_2022_1197_MOESM1_ESM.pdf]

Appendix for

An expert-curated global database of online newspaper articles on spiders and spider bites

Mammola S. et al.

Supplementary materials include:

1) **Supplementary text.** A "**STANDARDIZED PROTOCOL FOR NEWS RETRIEVAL**", listing the procedure for retrieving news and extracting information.

2) **Supplementary text.** A continuously updated "**FREQUENTLY ASKED QUESTIONS (FAQ)**" section as soon as new questions came in.

3) **Supplementary text.** A section with "**NOTES RELEVANT TO THE DETERMINATION OF Venom_error**", describing the most common envenomation symptoms to standardize the assessment of the errors related to spider venom of each news article. This section is largely based on Vetter & Isbister (2008) and other specialized literature.

4) **Table S1**

5) **Table S2**

6) **Figure S1**

7) **Figure S2**

8) **Supplementary literature.**

1 – STANDARDIZED PROTOCOL FOR NEWS RETRIEVAL

To ensure that different authors working in different countries and languages adhered to a univocal data mining strategy, we prepared a protocol beforehand for retrieving and extracting information from the news. This protocol was shared with all authors using Google Drive during the collection of data. The general protocol has been adapted from Mammola et al. (2020), which you can consult for more details.

a) Searching strategy

Searching for news is done using Google News. To be clear, this is not www.news.google.com; the searching is done within www.google.com and using the News tab as shown in Figure S1.

We are restricting the analysis to the period between **2010** and **2020.** In Google News, you can set the time interval for the search using *tools –> custom range*, as shown in Figure S2.

First, you will have to search the word "bite" (in your language), followed by the common name for spider (again, in your language). Repeat each search using "sting" instead of "bite", as it is frequently misused by journalists (e.g., "spider sting"). Depending on your country, repeat the search by changing the general word "spider" with latin and vernacular names of the main "dangerous" (medically important: e.g. *Latrodectus, Atrax, Loxosceles, Phoneutria*, etc. and/or widely feared: *Cheiracanthium, Lampona, Steatoda*, etc.) spider species in your country and nearby countries (Table S1–S2).

For each unique keyword search (e.g., "spider bite"), check up to the final available page in Google news extracting relevant news. You will focus on all media reports referring to one or more 'encounters' between humans and spiders.

Do include: i) All media reports referring to a human-spider encounter (e.g., a person talking to the local newspaper about the spider they found in their house; a man "bitten by a spider"; the lady who died due to a "spider bite"); ii) events that both occurred in your country or abroad (e.g., an Italian newspaper talking about a bite by a *Steatoda* that occurred in England have to be included).

Do not include: i) Media reports not reporting about a specific encounter between a human and a spider (e.g., news discussing best practices to heal from a spider bite);

ii) news published before 2010.

b) Information to extract

For each unique media report, extract the following information (note that the R notation 'NA' is used for missing data):

Url. The link to the online news.

ID. Assign an ID to each news item (e.g. "ITA_001", "ITA_002", etc.). **Important:** If a news article reports multiple human-spider encounters, please use a separate row for each encounter, repeating the news ID and all the information for each of these encounters (see "Example_1" in the template).

Language. Language used in the news.

Newspaper. Name of the newspaper (or news source if not a traditional paper).

Newspaper_type. "Traditional newspaper" (Official newspaper in your country, with both a printed and an online version), "Online newspaper", or "Magazine" (for magazine, tabloids, etc.)

Circulation. Circulation of the newspaper: "Regional", "National", "International".

Date of publication. y= year; m = month; d = day.

Title. Title of the article.

Species. Species' Latin name (as mentioned in the newspaper, even if clearly wrong). If the species is not clear, use "Gen sp".

Year_event. What year did the human-spider event take place?

Location_event. What city/region did the human-spider event take place? e.g. London. If unclear, use "NA" (missing data).

Country_event. Which country did the human-spider event take place? e.g., UK. If unclear, use "NA" (missing data).

lon (longitude) and **lat** (latitude). WGS84 decimal degrees coordinates of the

Location_event (e.g., 7.473268; 44.72862827). You can extract these from Google Earth / Google Maps.

Bite. Is a biting event described? 1 = yes; 0 = no.

Death. Was it a fatal biting event? 1 = yes; 0 = no

Sensationalism. Is the article sensationalistic? 1 = yes; 0 = no. This is subjective, but frequent words associated with sensationalistic content are: alarm (“allarme”), agony (“agonia”), attack (“attacco”), devil (“diavolo”), fear (“paura”), hell (“inferno”), killer (“assassino”), nightmare (“incubo”), panic (“panico”), terrible (“terribile”), and terror (“terrore”). Examples of titles of sensationalistic versus non sensationalist media reports focusing on the same Event_ID are, respectively: i) “[...] Sardinia and the nightmare of venomous spiders” versus “Black widow spiders spotted in Sardinia, but the expert is happy: it is an indicator of biodiversity”; ii) “Alarm in Rome: Violin spiders strike again and again. Boom of hospitalisations” versus “Bitten by a violin spider, he was immediately hospitalized”.

Figure_species. Is there a photo of the species? 1 = present; 0 = absent

Figure_bite. Is there a photo of a bite (or other injured body parts, e.g. an amputated leg)? 1 = present; 0 = absent

Expert_arachnologist. Was an expert capable of identifying spiders consulted in the news(arachnologist, entomologist, taxonomist, etc.)? 1 = yes; 0 = no.

Expert_doctor Was a doctor / medical professional consulted in the news? 1 = yes; 0 = no.

Expert_others Was some other potential “expert” consulted in the news? E.g. pest controllers. 1 = yes; 0 = no.

Taxonomic_error. Does the article contain a taxonomic error? E.g., the common mistake of calling spiders “insects” but also more subtle inaccuracies in terms of Linnean taxonomic ranks [e.g.,a report said:“…the ‘malmignatta’, a genus of Italian spider belonging to the family of the species of the black widow”]. 1 = yes; 0 = no.

Venom_error. Does the news contain errors in venom and other physiological aspects? 1 = yes; 0 = no.

Anatomy_error. Does the news contain errors in anatomical aspects? E.g., the spider stung its victims, the spider with 6 legs, etc. 1 = yes; 0 = no.

Photo_error. Error in the photographs of the species (the species in the media report does not correspond to the species mentioned in the text or if the attribution is not possible (e.g., blurry photographs). 1 = yes; 0 = no. If there is not a photo of the species use “NA”.

2 – FREQUENTLY ASKED QUESTIONS (FAQ)

List of frequently asked question that we compiled during the data mining strategy, so that all author could apply the same set of rule to resolve difficult cases.

1) Do we score articles where the spider bite/encounter is not the focus? Example: A story of a woman's legal issues and a one line reference to her going to a hospital for a spider bite. Example 2: An article that references someone who thought they were bitten by a spider but realize soon after that it is something else (e.g. bug bites)

Yes, but mention in the "notes" if they say that they later discovered it was not a spider.

2) Do we score articles referencing bites that occurred decades/centuries ago?
Ex. "McDonald thought it was a spider bite; she'd been bitten once by a brown recluse in Arkansas, where she'd grown up." In this case, two different bites would count for two data points, right?

Do not report those vague mentions, unless you are able to assign a specific date (1967) and locality (Arkansas). Only in the latter case will you have to replicate the news in the database, using the same news ID.

Example: A newspaper article reports a bite that occurred in Christchurch in 2016, but also mentions that the person who was bitten was also bitten when they were a child living in Sydney. Report the Christchurch event but not the Sydney event, because "when he was a child" is not a specific date.

Example2: A newspaper article reports a bite that occurred in Christchurch in 2016, but also mentions that the last bite related to that species occurred in 2010 in Timaru. In this case, report the Christchurch event, assign the ID to this news and then replicate the same ID to also include the event in Timaru (2010).

3) Are we considering people working at Occupational Safety and Health Administration or a City Environmental Preservation Division as experts?

Yes, in the category Expert_others.

4) If a bite occurs on the plane, should the location be where the plane departed from?

If the biting event occurs on an airplane, indicate it as follows:

Location_event: Flight Geneva – New York ; Country_event: Switzerland (i.e, The take-off place) ; lon: NA ; lat: NA

5) We aren't counting the description of a "poisonous spider" to be a venom_error, right? This is a very common error.

Calling a spider "poisonous" isn't a venom_error because it's a term that the vast majority of people use interchangeably with "venomous". More recently, there has been an uptick of biology folks who like to correct lay people on the differences between those terms. It's analogous to laypeople referring to bugs as all insects, whereas biologists will know that bugs are the common name of a certain order of insects. In any case, scoring it as a venom_error will likely cause half of the articles that focus on spiders to be scored as a 1, so that is something to consider carefully and specifically mention in the paper.

6) Are we calling black widows "*Latrodectus* sp."? I wasn't sure if we can infer the species from the U.S. even if they are being found in grapes from California given the range overlap.

If you are not sure, use Latrodectus sp. However, whenever you can assign with confidence a species, e.g. using indirect evidence on range, it is better to go to the species level (in the "notes" you can specify it was assigned based on geography).

7) We are not using blog posts, right?

We do not include blogs.

8) Would we consider articles to be committing taxonomic errors when they: 1) describe the spider event in one sentence and then 2) generalize by 'people who have insects in their ears should do X'. They don't explicitly call spiders insects, but an uninformed reader might infer this from recommendations on what to do with insect problems that are described next in the article.

Yes, it is a taxonomic error in this case.

9) Is it sensationalist to call a spider "deadly"?

Unless the story is about a spider species that has caused verified fatalities in the past, it is sensationalist, and even for Latrodectus (which to our knowledge has caused no fatalities for decades) it is leaning toward sensationalist ("dangerous" would be a non-sensationalist alternative).

10) What is the cut date for 2020 in terms of how recent articles should be included?

We began contacting people on the 1st of May 2020. A reasonable cut off would be up to 30th April 2020. Anyway, in any analysis on temporal patterns, we will probably end up omitting 2020 since it will not be a complete year, so feel free to include even news published after 1st of May.

11) For some reason my country is not included in Google news. What should I do?

In such a case, do all searches with regular Google. It is just a bit more laborious because you will get in a lot of unrelated (i.e. non-news) stuff and you can't set the date range, but ultimately it is the same.

12) When you say newspapers can this be any online media? For example, a major news source in NZ is Radio New Zealand or TVNZ which aren't technically newspapers, but they are online news sources that will then be fed into tv or radio news. I count these right?

Yes, include those.

13) Is circulation therefore relevant for these online sources as people from anywhere could access?

If it's in English but just a local radio it is a "National" circulation.

14) A few articles have used pictures of spider species that don't match the species they are writing about. However, if the photo caption clearly labels this other spider species correctly, would this be Figure_species =0, Error_Figure =0?

This is a tricky case. In the caption, do they say that the spider in the photo was the biting spider?

If so, go for Figure_species = 1 and Error_Figure = 1, because the reader will be misinformed that the spider species mentioned in the text look like the one

in the photo.

Otherwise, if they are reporting the photo for other reasons, use Figure_species = 1, Error_Figure = 0. In this case, there is a photo of a spider, but it is for another purpose and there is no mistake in its attribution.

15) If the article is written by a doctor and the subheading advertises the article as something like "why your spider bite might be something else, writes Dr. X", I assume Expert = 1?

Yes.

16) If the article is written by a professional outdoorsman/nature writer who was researching black widows, I assume Expert = 1? He was also the bite victim, but most of the articles on this event simply quote him when describing black widows.

Yes.

17) Should we use accompanying video material with news articles? Some of the news sources represent broadcasted TV news and the written articles are a shorter version of their segments. The videos often show spider pictures, bite pictures, and report taxonomic errors that are not in the written form. Example: https://turnto10.com/archive/man-bitten-by-rare-brown-recluse-spider

Yes, and in this case score Figure_species= 1 and Figure_bite = 1, since there is basically an iconographic element referring to the species.

18) There have been a series of articles following the bite of a celebrity who had necrosis supposedly after a spider bite. Only one article thus far states that the spider was a brown recluse and the ID was NOT attributed by the celebrity. Should I keep the spider species as "Gen sp." for all of the previous entries even if one article says he was bitten by a brown recluse? Other relevant factors are that 1) the spider was never found, 2) the article probably inferred the spider based on his symptoms, and 3) this incident happened in Los Angeles, which doesn't have brown recluses outside of a few infested buildings.

Yes, call it brown recluse in that specific one and use "gen sp" for the remaining news. Please, mention in the note if it is clearly wrong (e.g., for geographical reasons).

19) If the article is published in an NZ newspaper but is a direct reprint of an international story (in this case Daily Mail), do I still include it?

Yes.

20) An article says that the 'venom had eaten the flesh'....is this a thing that happens with brown recluse bites?

A venom error can be many different things, and depends on the species. Please refer to the "NOTES RELEVANT TO THE DETERMINATION OF Venom_error".

3 – NOTES RELEVANT TO THE DETERMINATION OF Venom_error

Spider envenomation symptoms and treatments

If a newspaper article describes symptoms of bites or envenomation treatments, these can be compared to the information below to determine whether they constitute a Venom_error. For example, if an article describes a black widow bite causing "necrosis" this would be considered a venom error. Similarly, a "recluse bite treated with antivenom" would be a venom error since there is no such antivenom in most cases – note, however, that there is an antivenom for *Loxoscelism* available in four Latin American countries.

This information is largely based on Vetter & Isbister (2008) (all quoted elements). Lopes et al. 2020 was also used for a recent summary of symptoms, diagnosis, and treatments for recluse bites.

a) Widow spiders – *Latrodectus* spp.

Envenomations are characterized by "pain (local and generalized) associated with nonspecific systemic effects, diaphoresis, and less commonly other autonomic and neurological effects."

<u>Symptoms:</u> "Puncture marks are uncommon due to the spider's small fang size."

"Pain patterns appear to vary among different species, with local and radiating pain predominating with *L. hasselti* bites in Australia, and abdominal, back, and chest pain more common in North and South America, South Africa, and Europe."

"Nonspecific systemic effects occur in approximately one third of the cases. Hypertension, agitation, fever, priapism, patchy paralysis, paraesthesia, fasciculations, and cardiac effects are less common. Muscle spasm is reported from some widow spider bites, including cases in North America. Envenomations by brown widow spiders, *L. geometricus*, are less severe and differ from typical latrodectism symptoms."

"The duration of latrodectism effects in untreated cases ranges from a few hours to days, with persistent pain being the major problem."

<u>Venom:</u> "α-latrotoxin is thought to be the major neurotoxin responsible for latrodectism."

Treatments: include antivenom & analgesia.

b) Recluse spiders – *Loxosceles* spp.

Symptoms: Envenomations can "cause severe skin lesions and rarely systemic effects; most bites are unremarkable."

"Bites can vary from mild, self- limiting erythema to large, necrotic ulcerations. However, severe effects are uncommon; in one study only 3 of 111 suspected *L. reclusa* bites required skin grafts."

"Four categories of loxoscelism exist: (*a*) no effects, (*b*) minor injury with edema and erythema, healing without supportive care, (*c*) dermonecrotic injury with development of a hardened ulcer that sloughs off, leaving a scar, and (*d*) systemic effects with hemolysis sometimes leading to disseminated intravascular coagulation and, in rare cases, renal failure and death, reported to occur mostly in children."

"In what is perceived as the typical dermonecrotic loxoscelism event, the bite is almost painless, if felt at all. Within 2 to 8 h, there is a sharp pain that progresses to a burning sensation. The bite site pales, with surrounding tissue becoming red and swollen. In lesions that become significant, a central, sinking, blue-gray wound develops that is sometimes accompanied by a characteristic bull's eye wound (red erythematous center, white ring of induration, outer blue cyanotic ring). During the first week, a central eschar may form and then slough off a few days later."

Venom: "the medically important component appears to be sphingomyelinase D"

Treatment*: "For most *Loxosceles* bites, RICE (rest, ice, compression, elevation) therapy is considered a proper remedy because most events are minor; therefore, conservative treatment is advocated in most cases."

*Often (misdiagnosed) "recluse bites" are treated with antibiotics. I would consider this a "venom error" if reported in a news article because necrosis is caused by venom, not bacteria. Bacterial infections such as MRSA are probably the most common conditions mistaken for spider bites.

c) Funnel-webs – *Atrax* spp. and *Hadronyche* spp.

"These are the world's most dangerous spiders according to animal lethal dose studies and clinical cases." Envenomation is "life-threatening and rapid in onset, and effective antivenom is available."

Symptoms: "Funnel-web spider bites cause local pain at the bite site, usually with obvious puncture marks. In a large proportion of cases, nothing further develops. Mild envenoming occurs in some cases with local neurotoxicity (paraesthesia, numbness, or fasciculations) and/or nonspecific systemic effects. Severe envenoming is characterized by autonomic excitation (hypertension, tachycardia or bradycardia, myosis or mydriasis, lacrimation, piloerection, and diaphoresis), neuromuscular excitation (paraesthesia and muscle fascicu- lations and spasms), nonspecific systemic ef- fects (abdominal pain, nausea, vomiting, and headache), pulmonary edema, agitation, and, less commonly, coma."

Treatment: "Funnel-web spider antivenom is the primary treatment and appears to be effective in all medically important species"

"Pressure immobilization bandaging is the recommended first aid and should remain on until the patient is transported to a hospital with antivenom."

d) *Phoneutria* spp.

Occasionally transported in bananas cargos.

Symptoms: "Most *Phoneutria* bites cause mild effects including pain, localized diaphoresis, and piloerection. Systemic envenoming is less common, characterized by stimulation of the parasympathetic nervous system manifesting as priapism in males and bradycardia and hypotension. This may be life-threatening, particularly in children and the elderly."

Treatment: "Antivenom therapy is available in Brazil, where the majority of cases occur but it is only used in a few cases (2.3% in the largest series)."

e) Wolf spiders – Lycosidae

Do NOT cause necrotic lesions.

f) White-tailed spiders – *Lampona* spp.

No evidence that they cause necrosis.

g) Black house spiders – *Badumna* spp.

Mild symptoms, no necrosis.

h) Yellow sac spiders – *Cheiracanthium* spp.

Have been incorrectly implicated in dermonecrosis. “Yellow sac spider bites are typically painful like a bee sting during fang puncture; minor symptoms include erythema, edema, and pruritus, resolving in a few days.”

i) Hobo spiders – *Eratigena agrestis,* previously *Tegenaria agrestis*

Wrongly associated with dermonecrosis in the Pacific Northwest of North America (see Gaver-Wainwright et al. 2011).

j) Other spiders

“Many other spiders, whose bites cause mild and transient effects, are commonly recognized and encountered worldwide.” These include ***Steatoda* spp.,** which can cause “minor latrodectism effects”.

4 – Table S1

List of “deadly” spiders. Medically important spiders that have caused fatalities/are potentially fatal (Isbister & Fan 2011; Vetter & Isbister 2008).

Taxon	Distribution range	World Spider Catalog (2021) link
Latrodectus	worldwide	https://wsc.nmbe.ch/genus/3502
Loxosceles	Americas; tropics and subtropics	https://wsc.nmbe.ch/genus/3087
Sicarius	South America	https://wsc.nmbe.ch/genus/3088
Atrax	Australia	https://wsc.nmbe.ch/genus/1061
Hadronyche	Australia	https://wsc.nmbe.ch/genus/1063
Phoneutria	Central & South America	https://wsc.nmbe.ch/genus/636

5 – Table S2

List of feared spiders. Spiders whose bites have more minor consequences and/or that are perceived to be dangerous—this will vary by country/region.

Taxon	Reference	World Spider Catalog (2021) link
Theraphosidae (Australia)	Isbister et al. 2003	https://wsc.nmbe.ch/family/100
Poecilotheria	Fuchs et al. 2014	https://wsc.nmbe.ch/genus/3413
Missulena	Isbister 2004	https://wsc.nmbe.ch/genus/2
Sparassidae	-	https://wsc.nmbe.ch/family/90
Dysdera crocata	Vetter & Isbister 2006	https://wsc.nmbe.ch/species/9736
Steatoda	-	https://wsc.nmbe.ch/genus/3542
Cheiracanthium	Vetter et al. 2006	https://wsc.nmbe.ch/genus/1950
Lampona (Australia)	Isbister & Gray 2003	https://wsc.nmbe.ch/genus/1107
Eratigena agrestis (North America)	Vetter et al. 2003	https://wsc.nmbe.ch/species/668
Badumna	Isbister & Gray 2004	https://wsc.nmbe.ch/genus/707
Lycosidae	Isbister & Framenau 2003	https://wsc.nmbe.ch/family/51

5 – FIGURE S1

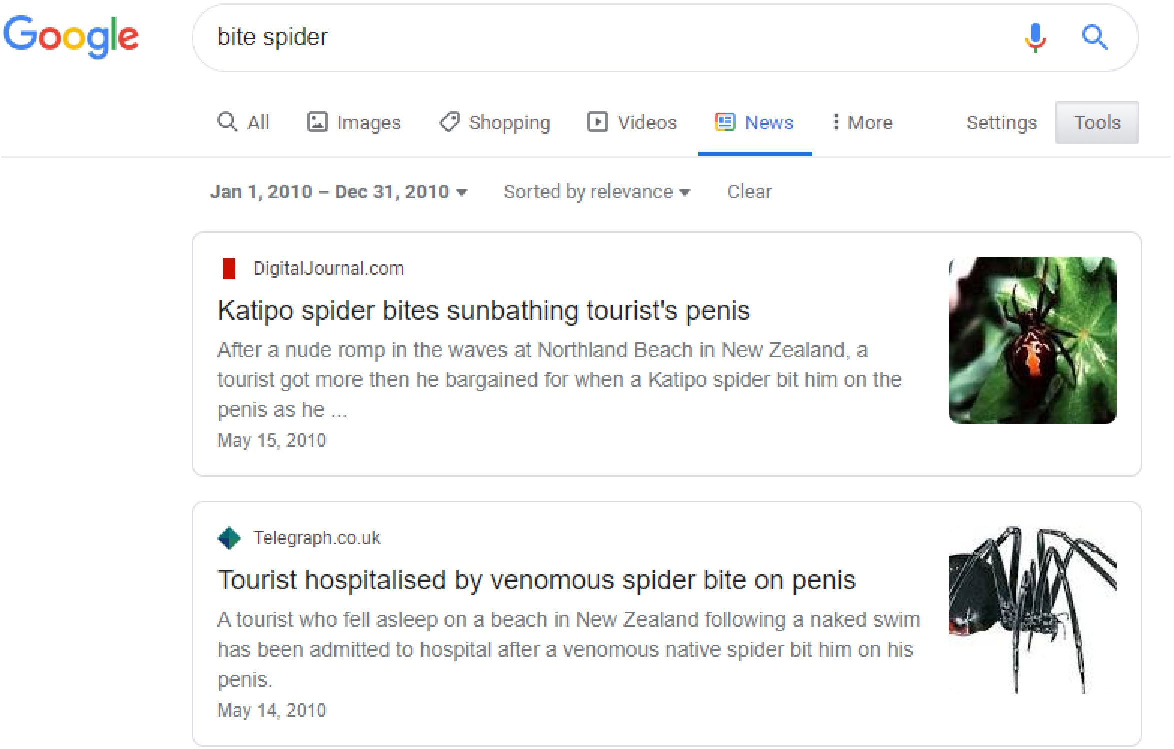

Screenshot of a Google page, illustrating how to access Google News.

6 – FIGURE S2

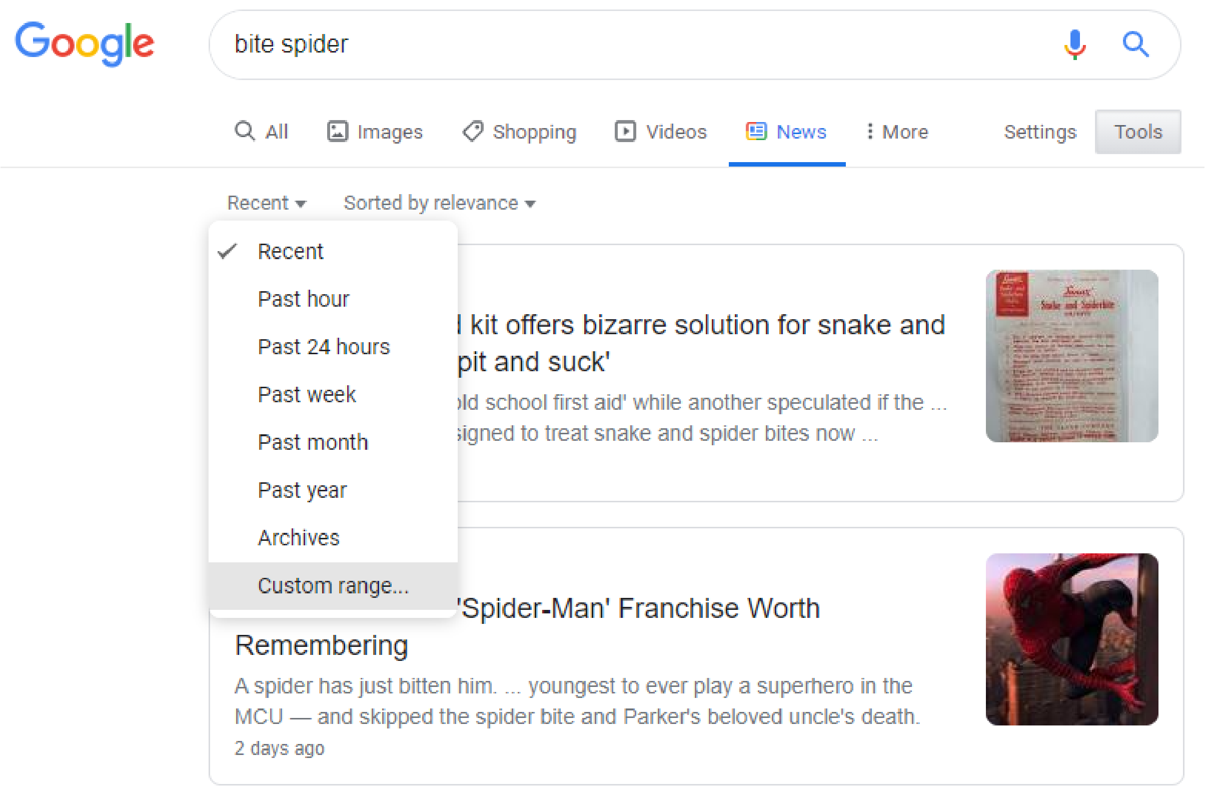

Screenshot of a Google page, illustrating how to set data range.

7 – SUPPLEMENTARY LITERATURE

Fuchs, J., von Dechend, M., Mordasini, R., Ceschi, A., & Nentwig, W. (2014). A verified spider bite and a review of the literature confirm Indian ornamental tree spiders (*Poecilotheria* species) as underestimated theraphosids of medical importance. *Toxicon* 77: 73-77.

Gaver-Wainwright, M. M., Zack, R. S., Foradori, M. J., & Lavine, L. C. (2011). Misdiagnosis of spider bites: bacterial associates, mechanical pathogen transfer, and hemolytic potential of venom from the hobo spider, *Tegenaria agrestis* (Araneae: Agelenidae). *Journal of Medical Entomology* 48(2): 382–388.

Isbister, G. K. (2004). Mouse spider bites (*Missulena* spp.) and their medical importance. *Medical journal of Australia* 180(5): 225–227.

Isbister, G. K., & Framenau, V. W. (2004). Australian wolf spider bites (Lycosidae): clinical effects and influence of species on bite circumstances. *Journal of Toxicology: Clinical Toxicology* 42(2): 153–161.

Isbister, G. K., & Gray, M. R. (2003). White□tail spider bite: a prospective study of 130 definite bites by *Lampona* species. *Medical Journal of Australia* 179(4): 199–202.

Isbister, G. K., & Gray, M. R. (2004). Black house spiders are unlikely culprits in necrotic arachnidism: a prospective study. *Internal Medicine Journal* 34(5): 287–289.

Isbister, G. K., Seymour, J. E., Gray, M. R., & Raven, R. J. (2003). Bites by spiders of the family Theraphosidae in humans and canines. *Toxicon* 41(4): 519–524.

Lopes, P. H., Squaiella-Baptistão, C. C., Marques, M. O. T., & Tambourgi, D. V. (2020). Clinical aspects, diagnosis and management of Loxosceles spider envenomation: literature and case review. *Archives of toxicology* 94: 1461–1477.

Mammola, S., Nanni, V., Pantini, P., & Isaia, M. (2020). Media framing of spiders may exacerbate arachnophobic sentiments. *People and Nature* 2(4): 1145–1157.

Vetter, R. S., & Isbister, G. K. (2006). Verified bites by the woodlouse spider, *Dysdera crocata*. *Toxicon* 47(7): 826–829.

Vetter, R. S., & Isbister, G. K. (2008). Medical aspects of spider bites. *Annual Review of Entomology* 53, 409–429.

Vetter, R. S., Isbister, G. K., Bush, S. P., & Boutin, L. J. (2006). Verified bites by yellow sac spiders (genus *Cheiracanthium*) in the United States and Australia: where is the necrosis? *The American Journal of Tropical Medicine and Hygiene* 74(6): 1043–1048.

Vetter, R. S., Roe, A. H., Bennett, R. G., Baird, C. R., Royce, L. A., Lanier, W. T., Antonelli, A. L., & Cushing, P. E. (2003). Distribution of the medically-implicated hobo spider (Araneae: Agelenidae) and a benign congener, *Tegenaria duellica*, in the United States and Canada. *Journal of Medical*

Entomology 40(2): 159–164.

World Spider Catalog (2021). World Spider Catalog. Version 22.0. doi:10.24436/2
